# Cardiopulmonary Exercise Test Methodology for Assessing Exertion Intolerance in Myalgic Encephalomyelitis/Chronic Fatigue Syndrome

**DOI:** 10.3389/fped.2018.00242

**Published:** 2018-09-04

**Authors:** Staci Stevens, Chris Snell, Jared Stevens, Betsy Keller, J. Mark VanNess

**Affiliations:** ^1^Workwell Foundation, Ripon, CA, United States; ^2^Department of Exercise and Sport Sciences, Ithaca College, Ithaca, NY, United States; ^3^Health and Exercise Science, University of the Pacific, Stockton, CA, United States

**Keywords:** functional capacity, stress test, oxygen consumption, post exertional malaise, functional impairment

## Abstract

**Background:** Concise methodological directions for administration of serial cardiopulmonary exercise testing (CPET) are needed for testing of patients with Myalgic Encephalomyelitis/Chronic Fatigue Syndrome (ME/CFS). Maximal CPET is used to evaluate the coordinated metabolic, muscular, respiratory and cardiac contributions to energy production in patients with ME/CFS. In this patient population, CPET also elicits a robust post-exertional symptom flare (termed, post-exertional malaise); a cardinal symptom of the disease. CPET measures are highly reliable and reproducible in both healthy and diseased populations. However, evidence to date indicates that ME/CFS patients are uniquely unable to reproduce CPET measures during a second test, despite giving maximal effort during both tests, due to the effects of PEM on energy production.

**Methodology:** To document and assess functional impairment due to the effects of post-exertional malaise in ME/CFS, a 2-day CPET procedure (2-day CPET) has been used to first measure baseline functional capacity (CPET1) and provoke post-exertional malaise, then assess changes in CPET variables 24 h later with a second CPET to assess the effects of post-exertional malaise on functional capacity. The second CPET measures changes in energy production and physiological function, objectively documenting the effects of post-exertional malaise. Use of CPET as a standardized stressor to induce post-exertional malaise and quantify impairment associated with post-exertional malaise has been employed to examine ME/CFS pathology in several studies. This article discusses the results of those studies, as well as the standardized techniques and procedures for use of the 2-day CPET in ME/CFS patients, and potentially other fatiguing illnesses.

**Conclusions:** Basic concepts of CPET are summarized, and special considerations for performing CPET on ME/CFS patients are detailed to ensure a valid outcome. The 2-day CPET methodology is outlined, and the utility of the procedure is discussed for assessment of functional capacity and exertion intolerance in ME/CFS.

## Background

A 2-day cardiopulmonary exercise test methodology (2-day CPET) was cited by the *Institute of Medicine* (IOM) (1) as a potentially useful tool to aid in the diagnosis and assessment of functional capacity in patients with Myalgic Encephalomyelitis/Chronic Fatigue Syndrome (ME/CFS). The IOM report concluded that ME/CFS is a neuroimmune pathology that affects multiple systems and contributes to exertion intolerance or an inability to recover normally following physical, cognitive or emotional exertion (1, 2). The IOM determined that “ME/CFS patients often have a level of fatigue that is more profound, more devastating, and longer lasting than that observed in patients with other fatiguing disorders” (1). The fatigue in ME/CFS differs from that experienced by controls and is unlike the fatigue associated with deconditioning. It is often described as “flu-like” and frequently includes “brain fog” or cognitive difficulties and other symptoms. This abnormal response to exertion is a hallmark symptom of ME/CFS referred to as post-exertional malaise (PEM). PEM is among the primary debilitating symptoms of ME/CFS, as well as fatigue-related impairment lasting more than 6 months, unrefreshing sleep, and usually cognitive impairment (brain fog) and/or dysautonomia. Muscle and/or joint pain often accompany these other symptoms, any of which could force a person with ME/CFS to stop work, avoid physical activity and, consequently, further reduce functional ability.

The lack of definitive biomarkers and no known cause or cure for ME/CFS contribute to patients suffering through medical misinterpretation, misunderstanding, and an overt bias toward characterizing the illness as a psychosomatic disorder. However, studies of exercise capacity reveal that a 2-day CPET procedure can provide evidence of the pathophysiology underlying the PEM that characterizes patients with ME/CFS (3–6). CPET methodology is standardized as a well-accepted procedure to assess physiological responses to exertion in many illness conditions (7). Adaptation of this valid, standardized and reliable procedure to assess abnormalities associated with PEM is particularly useful for identifying impairment in patients with fatigue-related illnesses. The purpose of this paper is to provide guidelines and helpful practices to applying CPET techniques in patients with ME/CFS.

### Cardiopulmonary exercise testing

Analysis of expired gases during an exercise test generates values that are useful in the assessment of functional capacity, illness severity, and illness characterization in ME/CFS. For instance, peak oxygen consumption (VO_2_peak) is a well-recognized objective indicator of functional capacity (8–11), and may be used to assess disease severity and predict coronary heart disease and all-cause mortality (12–14). Additionally, VO_2_peak provides a foundation to evaluate metabolic functional impairment. A CPET allows for the comprehensive and integrated analyses of cardiovascular, respiratory, metabolic and work indices to help discern the etiology of exertion intolerance in a growing population of patients with multiple chronic comorbidities (15). It can be applied similarly to better understand disease pathology in ME/CFS.

Classically, a single CPET provides physiological measures at rest and throughout incremental exercise to determine energy producing capacity at metabolically relevant time points including anaerobic threshold and peak effort. However, for ME/CFS patients, serial exercise tests are particularly useful to explore the unique post-exertional pathology associated with the illness. With a 2-day CPET, baseline functional capacity is determined with the first test, which also serves as a standardized stressor to elicit a post-exertional symptom flare. The second exercise test 24 h later provides a metric of change in physiological function due to the post-exertional response, and can indicate magnitude of impairment associated with a patient's compromised recovery. Doing the second exercise test 24 h following the first test allows comparison of performance capability without confounding influences of delayed onset muscle soreness. This serial CPET methodology is not unique to ME/CFS, and was reported previously to assess hormonal responses following an exercise stressor in overtrained athletes (16). Use of the 2-day CPET methodology in ME/CFS is of increasing interest for the same purpose; to assess a patient's ability to recover normally following exertion.

### CPET measures are reliable and reproducible

The 2-day CPET methodology is useful for assessing impaired recovery because CPET measures are readily reproduced in both healthy and diseased populations. Therefore, a failure to reproduce CPET measures on a subsequent test, despite peak effort on both tests, indicates a derangement of homeostasis.

#### Peak oxygen consumption

VO_2_peak is a highly reliable and objective measure of functional capacity (11, 17, 18). The reproducibility, or variability of this measure from one day to the next is also low. This is true across a broad population of healthy adults (11, 13, 17), children (19), and in those with pathologies such as heart failure (20–22), pulmonary hypertension (23), end-stage renal disease (24), cystic fibrosis (25), mild-moderate COPD (26), and stroke (27). Thus, VO_2_peak provides an objective measure of baseline functional capacity or maximal ability to produce energy for work. Failure to reproduce VO_2_peak during a second serial CPET, despite peak effort on both tests, implicates impairment of recovery mechanisms. This impaired recovery is consistent with PEM and suggests an underlying pathophysiology that contributes to an abnormal post-exertional state. Further, the magnitude of a test-retest decrement in VO_2_peak can be used to quantify the degree of impairment associated with PEM.

#### Anaerobic threshold

While VO_2_ at peak effort is an objective measure of maximum energy producing capacity; perhaps one of the most metabolically and functionally relevant transition points during an incremental exercise test is VO_2_ at anaerobic threshold. The VO_2_ and work intensity at anaerobic threshold are important indices of capacity to do continuous work, as activity above the anaerobic threshold is rapidly fatiguing and cannot be sustained. Anaerobic threshold is the exercise intensity at which the anaerobic contribution to energy generation is significant enough to cause non-linear increases in muscle and blood pH, lactate and carbon dioxide concentration. This transition is typically identified using serial measures of blood lactate obtained throughout an incremental exercise test to ascertain at which VO_2_ a non-linear increase in blood lactate occurs. The ventilatory stimulus of carbon dioxide causes a similar response in expired ventilation to that of blood lactate. This makes the ventilatory threshold a good non-invasive metric for the anaerobic threshold, which is referred to as the ventilatory anaerobic threshold (VAT).

Test-retest measures of oxygen consumption and work that correspond with VAT are stable over time with the same test modality, and vary within about 7–12% in both healthy individuals (5, 6, 18, 28) and a number of other pathological conditions (21, 22). Because VO_2_@VAT is a reliable and reproducible measure, a reduction in VO_2_@VAT over serial exercise tests indicates an underlying limitation in the capacity to meet daily energy demands via aerobic energy production.

### Functional capacity of ME/CFS patients

Peak oxygen consumption of ME/CFS patients obtained during a single CPET has been used to characterize functional status in adults with ME/CFS (5, 29–35) as well as adolescents (36, 37). However, patients and/or physicians typically seek this type of assessment after an individual has been physically inactive or low active for at least 6 months. Because of this it is often argued that low VO_2_peak in a patient with ME/CFS is due simply to physical deconditioning.

Compared to healthy controls, VO_2_peak of adults with ME/CFS varies from about 30-91% of predicted values for age and sex (5, 30–35, 38). This compares to values for adolescents with ME/CFS between 86 and 90% of predicted VO_2_peak for healthy controls of similar age and sex (36, 37). While low, values for some ME/CFS patients may be consistent with physical deconditioning, and may not be considered clinically remarkable. Consequently, VO_2_peak measured during a single CPET does not necessarily provide objective evidence of impaired recovery or PEM, whereas PEM is a cardinal symptom of ME/CFS, and management of PEM is a primary goal of treatment. Therefore, provocation of PEM may be accomplished using a standardized stressor of a single CPET. However, quantifying functional decrement due to PEM following the first CPET requires a second CPET administered 24 h later to determine if the patient can reproduce CPET measures within normal test-retest variability. Assessing the severity of PEM is useful for treatment for ME/CFS, and also provides objective evidence of impairment for purposes of disability review. The balance of this paper will detail the effects of PEM on recovery in ME/CFS and special considerations for testing those with ME/CFS for a valid and objective assessment of functional capacity and exertion intolerance.

## Results

### Studies of 2-day CPET in ME/CFS

Studies of exertion intolerance in ME/CFS using a 2-day CPET methodology indicate an impaired ability of patients to reproduce CPET results. Several studies have shown that ME/CFS patients are unable to reproduce values for VO_2_peak on serial CPETs (5, 6), VO_2_ at ventilatory threshold (6), and/or peak workload, or workload at ventilatory threshold (4). Additionally, abnormal responses to exercise such as hemodynamic or ventilatory responses may be observed in ME/CFS patients. Abnormalities in hemodynamic and ventilatory responses may or may not appear in some patients during the first exercise test, whereas others only display abnormalities during CPET2 following the onset of PEM.

Values for VO_2_, work rate and heart rate obtained at the VAT can also be examined for reproducibility. For example, test-retest VO_2_@VAT values are reliable and reproducible in both normal subjects and in various pathological conditions (22). But ME/CFS subjects often fail to reproduce values measured at VAT (3, 4). Compared to VO_2_peak, VO_2_@VAT is more indicative of the capacity to perform activities of daily living. Sustained activity at intensities that exceed VO_2_@VAT will eventually result in fatigue (15). Measures that coincide with VAT are important indicators of metabolic impairment and delayed recovery in ME/CFS patients. The failure to reproduce measures that correspond to VAT may be useful for identifying metabolic anomalies of energy production, and describing the magnitude of impairment due to PEM.

## Discussion

### CPET1 to CPET2 decrement indicates post-exertional malaise

The post-exertional effect on energy production are signaled by changes in values measured across two CPETs. Diminished responses, or abnormal changes in metabolic, cardiac or hemodynamic measures during incremental exercise indicate impaired recovery due to muscular, cardiovascular, pulmonary or autonomic dysfunction. Changes in these values from CPET1 to CPET2 should be compared to normal ranges for test-retest reproducibility. Abnormal variability between tests is evidence of impaired recovery where both symptoms and changes in CPET values represent abnormal perturbation. An assessment of 2-day CPET data should include comparisons with normal age/sex values, and between CPETs for peak and VAT measures of VO_2_, work output, heart rate, blood pressure, minute ventilation, oxygen saturation, as well as transitional (rest to peak) changes in all measures. Additionally, the V_E_/VCO_2_ slope or lowest value during a CPET should be scrutinized for ventilatory decompensation. The magnitude of change from CPET1 to CPET2 is considered abnormal if in excess of normal test-retest variability. CPET measures and normal test values appear in Table 1. Additionally, patient reports of symptom flares with PEM following a CPET further support the pathological recovery response to exertion evidenced by the 2-day CPET results.

**Table 1:** Typical CPET values of intrest for ME/CFS patients.

**CPET variables**	**Description/significance**	**Normal** **values/response**	**References**
Peak VO_2_	-Highest VO_2_ obtained during exercise -indicates biological functional capacity	Wide range by age, sex, fitness level % predicted value should be 85-100%	(11)
VO_2_@VAT	-Submaximal VO_2_ -occurs at point of dislinear increase in V_E_ -generally associated with anaerobic threshold -represents upper limit of workload that can be sustained for prolonged period	45–65% peak VO_2_	(39)
Peak RER	-ratio of VCO_2_/VO_2_ -best non-invasive indicator of exercise effort	>1.1-maximal effort 1.0–1.1-good effort <1.0-poor effort	(40)
Ve/VCO_2_ slope@VAT; Ve/VCO_2_ slope@RCP*; Lowest Ve/VCO_2_	-Indicates ventilatory efficiency and matching of ventilation to pulmonary perfusion	Generally <30, however normal values are age and sex dependent	(41)
PetCO_2_	-also represents matching of ventilation and perfusion and cardiac function	Rest: 36-42 mmHg From rest to VAT, increases 3–8 mmHg Decreases following VAT intensity	(42)
O_2_pulse	-ratio VO_2_/HR -indirect indicator of cardiac work	Continual linear rise thru exercise with possible plateau approaching peak effort	(43)
Peak heart rate	-highest HR during CPET -in patients not prescribed beta blockers provides insight into chronotropic competence and cardiac response to exercise -peak HR should not be used as primary indicator of subject effort given its wide variability	Chronotropic incompetence is ≤ 85% age-predicted heart rate reserve	(44)
HR recovery@1 min post peak effort	-Difference between peak HR and HR@1 min into recovery -provides insight into parasympathetic reactivation	Should have >12–18 bpm recovery in 1st min following peak exertion	(15)
Exercise BP	Provides insight into CV response to exercise and left ventricular afterload	During exercise SBP should increase 10 mmHg/3.5 ml^.^kg^−1.^min^−1^ VO_2_; DBP should not change >±10 mmHg from rest	(45)
SpO_2_	-non-invasive indicator of arterial hemoglobin saturation	>95% at rest and throughout exercise	(46)
ECG	-rate, rhythmicity and perfusion of the heart	Minimal waveform changes, no significant deviation from normal sinus rhythm	(47)
Subjective symptoms	-to determine subject perception of symptoms limiting exercise -Rating of Perceived Exertion (RPE) -dyspnea scale -pain scale	Limiting factor is muscular fatigue with no significant difference in dyspnea, pain	(48, 49)

### Other CPET measures

In addition to VO_2_, other measures should also be assessed both within and between CPETs to confirm normal responses to incremental exercise including hemodynamic, ventilatory variables, and work rate measures. Abnormal responses to incremental exercise for heart rate, blood pressure, minute ventilation (V_E_), workload (e.g., VO_2_/work) and temperature implicate specific aspects of energy production and physiological systems affected by ME/CFS which may contribute to PEM. Immediate post-test recovery measures (e.g., HR, BP, ECG, O_2_sat, recovery time) should be closely monitored as well to determine normal post-CPET recovery responses. Post-test recovery dynamics should also be compared between CPET1 and CPET2. Disrupted post-test recovery dynamics, particularly following CPET2, are not unusual in this population. Signs and symptoms should be documented before during and after exercise, including pain and dyspnea.

### VAT is highly relevant for an ME/CFS patient

The majority of daily energy demand is met via aerobic metabolic processes, which typically provides energy for daily activities such as normal speed walking, seated tasks, and other activities of daily living. A reduction in VAT following exertion in persons with ME/CFS may force reliance on anaerobiosis to support lower intensity work and subsequently lead to premature fatigue. From a practical standpoint, a reduction in the workload at which VAT occurs is believed to be consistent with the post-exertional decrease in function that coincides with PEM in ME/CFS patients. For example, with PEM induced by CPET1, it is not uncommon for patients to present with anaerobic predominance (early anaerobiosis), even during seated rest at the start of CPET2. Functionally, such patients rely on energy produced via anaerobic metabolism simply to perform resting and/or low level activities. It is not surprising when fatigue occurs under these circumstances.

To determine the point at which VAT occurs during a CPET, there may be several algorithms in the software of a metabolic measurement system to identify the VAT breakpoint. Perhaps most notable, the V-slope method, originally described by Beaver, Wasserman and Whipp (50), makes use of the relationship between minute ventilation (V_E_) and ventilatory removal of carbon dioxide (V_CO2_) during incremental exercise to determine the VAT. For consistency, the same algorithm should be used to identify VAT for both tests within the 2-day CPET method. An additional concern when testing ME/CFS patients is how a potentially abnormal ventilatory response may impact the determination of VAT. For this reason, VAT identified by an algorithm should always be scrutinized by a person(s) familiar with the determination of VAT to ascertain agreement with the algorithm-derived VAT.

## Methodology

### Exercise testing considerations for ME/CFS patients

The objectives of the 2-day CPET method are to; (1) assess VO_2_peak and VO_2_ at VAT during the first CPET, in addition to other test variable kinetics, and (2) compare measures from CPET1 and CPET2 to assess test reproducibility and normality of recovery response following CPET1. To ascertain the magnitude of change in CPET2 due to CPET1, it is critical that the ME/CFS patient begin the test in a baseline state representative of the patient's well-rested capacity. Characteristics unique to ME/CFS patients require special pre-test preparations that should be addressed beginning as early as 2–3 weeks prior to a scheduled 2-day CPET. The objective is to minimize pre-fatigue and PEM in a patient who is preparing to travel in order to complete the 2-day CPET.

#### Pre-test considerations

Factors such as travel to the test site, immediate pre-test (day of or even day before) paperwork that taxes cognitive function, and prolonged time in a common waiting area, even if seated, can all contribute to pre-test fatigue. Fatigue and PEM are exacerbated by physical, cognitive and emotional stressors (1), so every effort should be made to reduce such stressors where possible. Likewise, many ME/CFS patients experience hypersensitivity to light, noise, temperature, odors, and/or chemicals, so it is helpful to minimize environmental stimuli and maintain a generally low level of activity in the waiting area and testing environment.

Pretest directions/instructions should be in writing and given to the patient at least 1–2 weeks prior to arrival at a clinic. Included in these materials should be a clearly written pre-test checklist to assure that the patient adheres to pre-test preparation instructions (e.g., alcohol, caffeine, exercise and food restrictions prior to CPET, appropriate attire, etc.). Directions to the facility should include availability of disabled parking close to the building, and clear directions to the elevator or other lift assist as needed. Stairs (up and down) and long walks to the clinic should be avoided if possible as this will pre-fatigue the patient. It is reasonable to ask the patient prior to arrival if wheelchair assistance is indicated. Likewise, it is essential that the patient understands the importance of not becoming fatigued prior to the test, and plans travel to the test site with that in mind. When the test site is more than 1 h away, if feasible the patient should be encouraged to arrive the day before the scheduled test and spend the night locally. For some patients, 2 days of rest following air travel to a clinic may be necessary. It is essential that patients understand they should not drive a motor vehicle away from the clinic following either CPET, and plan accordingly. These recommendations may limit patient accessibility to testing, but should be considered to optimize quality of CPET data and patient safety.

#### Pre-test forms/questionnaires

Forms and questionnaires should be sent to the patient at least 2–3 weeks prior to a scheduled test. Completion of forms can be cognitively taxing for a person with ME/CFS and contribute to PEM, so sufficient time should be allowed for completion and return of forms to the clinic. In a clinic environment where a physician is present only part-time, prior arrangements are necessary to provide medical supervision during the 2-day CPET when testing a patient that meets criteria for high risk (7, 45). Similarly, sufficient time is necessary for the patient's physician to complete and return the referral form prior to testing the ME/CFS patient. Information provided to the patient should include explicit pretest instructions. Patients who experience cognitive impairment may be unable to process and respond quickly to copious or complex information, so providing simple, easily understood documentation helps improve adherence to pretest instructions. Paperwork that should be sent to the patient 2–3 weeks prior to a scheduled test may include the following:

- General information about test, payment options, clinic contact information
- Directions to clinic/parking, elevator, etc.
- Area lodging information, indicating hotels that provide shuttle service to your clinic
- Physician consent form
- Medical/health history form
- Informed consent document
- Fatigue status questionnaire; e.g., Bell Fatigue Scale (51), Short Form 36 Health Survey (SF-36.org), Multidimensional Fatigue Inventory (52), Fatigue Impact Scale (53)
- Listofmedications/non-prescription medications/supplements
- Day of test instructions (what not to eat/drink, appropriate clothing, etc.)
- Release of information form
- Recovery strategies/aids

#### Medications/supplements

The use of medications prior to testing must be clarified with the patient. If the purpose of testing is for clinical evidence of impairment or assessment of PEM, medications, including OTC medications, and supplements should be taken as prescribed, and at the same time of day prior to each CPET. However, for both research purposes and clinical diagnosis, limiting medications would be determined after consultation with the referring physician.

#### Test-day considerations

-Seek to minimize time in the waiting area prior to preparations for a CPET. A place to recline or semi-recline is helpful for a waiting patient, or when reviewing or clarifying pretest paperwork and procedures with the patient.

-Provide water throughout testing, and following CPET2, electrolyte replacement beverages can be helpful. Many ME/CFS patients have orthostatic intolerance so maintaining hydration with fluid and electrolytes (e.g., coconut water, sport drink) following CPET2 is helpful for expediting recovery. There are a number of anecdotal reports of plasma volume or salt loading reducing recovery time. Patients may consider arranging with their physician for a prescription of 1 L of IV normal saline infusion following completion of the 2-day CPET. However, if possible, there should be no intervention between the two CPETs.

#### Pretest procedures

-CPET1 session should begin by explaining the entire test day procedure in detail. Prior to obtaining informed consent, respond to all patient questions.

-Review the completed pretest forms, and seek clarification of information if necessary. Additional questionnaires or procedures (e.g., title table test, cognitive tests, lung function measures, etc.) should be limited to minimize pre-fatiguing the patient. Body weight should be measured (not self-reported) prior to each CPET, and height should be measured before CPET1. After 5 min of supine rest, obtain resting heart rate, blood pressure, O_2_ saturation, temperature, and monitor ECG. Monitoring the ECG throughout exercise testing is important for determining rate, rhythm and potential ischemia of cardiac tissue. Since many patients experience orthostatic symptoms, it is important to take measures after 5 min of supine rest and also during seated rest on the cycle ergometer prior to exercise.

-Explain use of the facemask or two-way valve to collect expired gases while the patient is supine. At this time, introduce and explain the Rating of Perceived Exertion (RPE)/Borg scale (48). Specify anchor intensities of 6 and 20 (or 0 and 10 for the modified scale) as the lowest and highest ratings of perceived exertion.

-Pain and dyspnea scales should be explained at this time as well. The seat height of the cycle ergometer should be adjusted to fit the subject and recorded, and the subject allowed to pedal at 0 Watts for a short period (<1 min). This will help the subject feel comfortable on the ergometer and reduce anxiety. To ensure safety during and after the CPET the patient should be monitored closely for adverse effects with continuous measures of blood pressure, oxygen saturation, ECG, and other indicators of stress.

#### Test modality

A cycle ergometer is preferred to a treadmill for CPET testing of ME/CFS patients. While walking is a familiar activity for most and involves larger muscle groups, a cycle ergometer allows for easy quantification of work output and metabolic cost of exercise. There is less noise artifact for cardiac monitoring and measurement of blood pressure on a cycle ergometer, and fluctuation in work output from holding treadmill handrails, and biomechanical efficiency is less of a concern. Additionally, problems with balance and instability in some patients, particularly when close to maximum effort, are minimized when using a cycle ergometer. Patients generally feel more secure and comfortable on a cycle vs. a treadmill. Although VO_2_peak may be 10–15% lower in healthy individuals when measured using a cycle ergometer compared to a treadmill, the benefits of safety and security on the cycle ergometer for the ME/CFS patient outweigh the risks associated with treadmill exercise for this population (54). Lastly, for accurate interpretation of test-retest findings it is critical to precisely reproduce the CPET1 workload protocol during CPET2, which can be accomplished more readily with the cycle ergometer. Ideally, an electronically-braked cycle ergometer affords the most accurate workload measures, and also provides smooth workload transitions for the patient. Cycle seat height should be the same for both CPETs.

#### Test protocol

Selection of the test protocol should be matched closely with the anticipated ability of the patient. The goal of the test protocol is to incrementally challenge energy production such that the patient is able to complete at least 8 min but no more than 12 min of cycling (45). For *moderately ill* ME/CFS patients who complete this protocol, workload increments of 10–15 W/min, beginning at 0 watts, is appropriate to achieve an 8–12 min test to maximum effort duration. However, for a patient with a significant history of physical training, a 20 or 25 W/min protocol may be appropriate. The same protocol should be used for CPET1 and CPET2. Typically, the following cycle ergometer protocol is used for testing the ME/CFS patient:

- **START**: 3 min seated rest on cycle—monitor ECG, VO_2_, and record BP and O_2_ saturation at min 2.
- **EXERCISE PROTOCOL**: first minute exercise stage—begin at 0 watts (no prior warm-up) and increase 10–15 watts per min, or as appropriate.
- **DURING EXERCISE**: Measure BP/O_2_ saturation/RPE every 2 min (e.g., @ 15W, 45W, 75W, etc.).
- **PEAK EXERCISE**: Obtain RPE, HR, BP at peak or immediate post exercise
- **POST EXERCISE**: Recovery measures of BP, HR/ECG, O_2_sat @ minutes 1, 3, 5, etc. until recovery when HR is within 20 bpm above pretest HR, close to pretest BP, normal ECG, asymptomatic.
- **CONFIRM** reason for test termination with patient.

Test termination should comply with testing guidelines (45) and is indicated by attainment of maximal effort, or test termination due to patient safety. When testing for evidence of disability, insurers and independent medical examiners will closely scrutinize patient effort. Therefore, criteria for maximal effort should be reported which could include; plateau in oxygen consumption with increases in workload, RPE ≥ 18 (6–20 scale), respiratory exchange ratio (RER) ≥ 1.1, or peak blood lactate ≥ 8 mM. These criteria support evidence of maximum effort during CPET. The RER criterion is generally considered a more valid indicator of patient effort compared to the other indicators (55). Generally, satisfying two of three criteria is acceptable to determine that maximum effort was given by the patient (56). However, it would be inappropriate and unethical to prime the patient regarding effort criteria, therefore, consistency of procedures and patient motivation during both CPETs should be maintained for a valid comparison between CPETs.

#### Patient risk

As with any maximal effort CPET, risk is conferred to the patient in completing such a test. Risk reduction is mitigated through standard procedures that include obtaining a relevant health history and completed cardiovascular disease risk questionnaire, a physician referral for testing, and clarifying with the patient any signs or symptoms suggestive of cardiovascular, pulmonary or metabolic diseases. Standardized guidelines for exercise testing are available and should guide decision making regarding risk classification of a patient and the need for medical oversight when conducting a CPET (7, 45, 57). When testing ME/CFS patients, it is not uncommon to find they may be well-screened for cardiovascular, pulmonary or metabolic disease risk in the course of trying to obtain a diagnosis of their illness symptoms. Due largely to the lack of knowledge and understanding of ME/CFS by medical professionals, average time from onset of ME/CFS symptoms to diagnosis is greater than 1 year, but 29 percent of patients surveyed reported that receiving a diagnosis took longer than 5 years (58, 59). Throughout efforts to obtain a diagnosis, patients commonly, but not always, visit and are screened by internists, cardiologists, rheumatologists, and others. Yet, there is insufficient evidence among this patient population to fully understand the relative risk to ME/CFS patients who complete the 2-day CPET procedure. As well, there is no evidence to suggest that the risk of untoward cardiovascular events varies from the general population. Due to the fact that many patients are well-screened prior to CPET testing, it could be suggested that risk for such an event may even be less than that of the general population. For ME/CFS patients, the greater concern of performing an exercise test is that associated with the potential for exacerbation or worsening of their typical PEM symptom profile. Because the disease is cyclic in nature, with patients often experiencing periods of remission and reactivation, and due to insufficient data on CPET testing in sufficiently large numbers of patients, it is unknown if performance of an exercise test, either submaximal or maximal, could worsen the overall illness status. Exacerbation of PEM symptoms via exercise is an inherent risk but also central to the efficacy of the 2-day CPET methodology, which should be acknowledged in the informed consent document. Most patients are well aware of their PEM symptom complex and the temporal expression of symptoms. However, particularly in patients who recently became ill, the exacerbation of symptoms due to exercise testing may coincide with the natural cyclic progression of the illness, and result in more severe and longer exacerbation of symptoms than is typical. Hence, it is difficult to predict the extent to which symptoms may flare and for how long. Patients should be fully aware of this risk prior to consenting to an exercise test. Strategies to minimize pretest energy expenditure during travel to a test site, preparation for an exercise test, and mitigating posttest symptom exacerbation are listed in Table 2.

**Table 2:** Strategies to provide ME/CFS patients for testing.

**TRAVEL**
1. Avoid waiting in long security lines. Call the airline for a wheelchair in order to conserve energy and bypass the line. Be sure to check in at skycap and they will have a wheelchair waiting. 2. Preboard the flight for extra time to store belongings. 3. Sign up for TSA pre check. The security lines are shorter and there is no need to remove shoes and computers from carry-on luggage. 4. Travel with noise canceling headphones, earplugs or both. 5. Bring an eye mask and travel pillow to make the trip more restful. 6. Cover your face with a mask to avoid unwanted germs. 7. Bring healthy snacks. 8. Wear compression socks or compression calf sleeves to promote circulation and reduce fatigue. 9. Travel to your destination a day early and take a day or more to rest if needed.
**HOTEL**
1. Ask for a quiet room away from the ice machine. 2. Bring earplugs. 3. Use a white noise app. 4. If possible use a shower bench or sit down while showering. 5. Use a robe to dry off after showering. 6. Stay hydrated. Buy a bottle of water. If the hotel has a fitness center they usually have filtered water for free refills and often have fresh fruit.
**GENERAL RECOVERY**
1. Take a warm bath with Epsom salts. 2. Stretch sore muscles slowly but frequently. 3. Pace activities by planning rest breaks during the day. 4. Use diaphragmatic breathing to promote relaxation and recovery. 5. Rest until recovered.

#### Calibration and quality control

Calibration of the metabolic cart is essential prior to and following each CPET to assure accuracy and validity of data. Of equal importance, is biological validation for long-term stability of metabolic cart accuracy.

Quality Assurance - Biological validation for quality assurance is also essential for valid data. Reproducibility of gas exchange measurements requires consistent testing methodology. Biological quality control can identify error not detected by automated calibration of the metabolic measurement system (60). Even when automated system calibration appears accurate, results may be erroneous (61). Biological validation can be achieved by testing laboratory staff on a monthly basis at matched submaximal work rates, and for VO_2_/work rate slope (62).

In general, routine maintenance based on the manufacturer's recommendation is essential for internal validity of data from the metabolic measurement system and electronic cycle ergometer. The validity of comparison of CPET1 and CPET2 data relies on valid and reliable measurement devices.

Software Considerations - Data sampling, averaging, graphical and summary reports are determined by software supplied with metabolic measurement systems. Sampling differences can greatly affect test results. Breath by breath measurements averaged over 15–20 s intervals will reduce the effect of random noise and improve data consistency. It is essential to display data in a tabular time down format with rest, start of exercise, and peak exercise clearly delineated. Peak values should be selected from data in time down format following visual inspection of data for overt outliers due to coughing, gagging, sneezing or talking. Only data from start of exercise to peak exercise should be used to determine VAT.

## Conclusions

Understanding and treating ME/CFS patients is hampered from a lack of diagnostic markers, heterogeneity in patient presentation, waxing and waning of symptoms within an individual patient, poor understanding of disease pathology, and the need to exclude other conditions. Cardiopulmonary exercise testing can provide helpful insights into this disease by better characterizing the unique post-exertional pathology of the illness.

Studies using one CPET-only are useful to elucidate immune activity and/or gene expression in ME/CFS because strenuous activity is known to induce considerable physiological changes similar to those associated with trauma (29, 63). As a quantifiable stressor, CPET has the capacity to reveal abnormalities across multiple systems that may not be apparent at rest by assessing the integrated response to exercise through comprehensive evaluation of the pulmonary, cardiovascular, haematopoietic, neuropsychological and musculo-skeletal systems (15). The inclusion of CPET could also be a primary consideration when designing clinical trials with functional endpoints (61). Determination of the respiratory exchange ratio, a measure exclusive to analysis of expired gases, provides the most accurate and reliable gauge of subject effort. This avoids problems associated with use of age-predicted maximal heart rate, which varies significantly in the general population and can be affected by both medication and pathology. Issues of response bias in self-report indictors of effort are also avoided. In the case of ME/CFS, the respiratory exchange ratio enables direct comparison between patient and control with confidence that both subjects were exposed to equivalent levels of physiological stress. Single CPET studies are also be useful for objective measurement of illness severity, pathophysiology, and for monitoring illness progression.

By comparison, the 2-day CPET methodology is useful for describing ME/CFS pathology as it provides objective and measureable changes due to impaired recovery across the two exercise tests. When the first test is conducted with a well-rested subject in a non-exacerbated state, this methodology allows for the characterization and quantification of post-exertional effects on functional capacity. Effects may be identified to correspond specifically to exercise at VAT or peak effort. Information gleaned from a 2-day CPET also offers objective evidence of impairment attributable to the effects of PEM, helps with patient management, informs therapeutic interventions, and tracks illness progression. Standardizing the 2-day CPET methodology to assess ME/CFS and other fatiguing illnesses across testing sites and study groups will allow for valid and relevant between-study comparisons. The goal is to better understand how impaired recovery impacts energy production and function, and hopefully determine the underlying pathophysiology of PEM as a disease component of ME/CFS.

## Author contributions

SS designed the article and contributed to writing the manuscript and editing drafts. CS contributed to writing the background section and editing. JS, BK, and JV contributed to writing the manuscript and editing drafts.

### Conflict of interest statement

The authors utilize CPET testing as a disability evaluation tool for which they charge a fee.

## Abbreviations

BP: Blood pressure
CPET: Cardiopulmonary exercise test
ECG: Electrocardiogram
HR: Heart rate
ME/CFS: Myalgic Encephalomyelitis/Chronic Fatigue Syndrome
O_2_pulse: oxygen pulse
PEM: Post-exertional malaise
RPE: Rating of perceived exertion
SEID: Systemic Exertion Intolerance Disease
VAT: Ventilatory anaerobic threshold
V_CO2_: Ventilatory removal of carbon dioxide
V_E_: Minute ventilation
VO_2_peak: Maximal oxygen consumption
VO_2_@VAT: oxygen consumption at ventilatory anaerobic threshold
VT: Ventilatory threshold.
